# Papillomavirus Infection and Prevention: How Much Does the Sicilian Population Know? An Observational Study

**DOI:** 10.3390/ijerph191711032

**Published:** 2022-09-03

**Authors:** Barbara Verro, Salvatore Gallina, Carmelo Saraniti

**Affiliations:** Division of Otorhinolaryngology, Department of Biomedicine, Neuroscience and Advanced Diagnostic, University of Palermo, 90127 Palermo, Italy

**Keywords:** human papillomavirus, papillomavirus vaccines, oral cancer, oropharyngeal cancer, health communication

## Abstract

**Highlights:**

- To date, incidence of HPV-related oral and oropharyngeal carcinoma is increased;
- Spread of HPV infection among youth is due to sexual precocity and promiscuity;
- Two HPV strains are primarily related to cancer onset: 16 and 18;
- There are different HPV vaccines but knowledge and adherence are still quite low;
- Not only young women must be aware on risk factors and vaccine.

**Abstract:**

Human papillomavirus is a sexually transmitted virus that is responsible not only for uterine cervical cancer, but also for the oral and oropharyngeal cancers. In this latter case, the virus indiscriminately affects both sexes at any age. Vaccination plays a key role in preventing infection and its possible consequences. Therefore, this study aimed to assess the degree of awareness of papillomavirus infection and its possible prevention in the Sicilian population. A prospective observational study was carried out on the Sicilian population through a self-administered questionnaire, consisting of 10 multiple-choice questions. The survey lasted from June 2021 to December 2021. A total of 844 respondents were included: 60.90% were female and 53.55% were aged between 21 and 30 years. Most of the surveyed population knew about the virus and the existence of the vaccine, however, many of them did not know about virus’s effects on the oral and oropharyngeal sites (49.17%), or about the indication for vaccination for males (39.69%). Oral and oropharyngeal papillomavirus-related cancers are an increasingly frequent finding, especially in young adults. Therefore, it is necessary and quite mandatory to educate the population about the risks that certain voluptuous habits may cause, with the help of general practitioners, schools, social media, and social networks.

## 1. Introduction

Human papillomavirus (HPV) is a sexually transmitted virus and is the leading cause of uterine cervical cancer [1]. In recent decades, its growing main role in the onset of oral and oropharyngeal carcinoma (OPC) was studied [2]. In fact, there is a reduction in the incidence of OPC due to smoking and/or alcohol. Nevertheless, there is an increased incidence of HPV-related OPC [3]. In 2017, De Martel et al. observed that about 30% of oropharyngeal cancers were HPV-related, with 29,000 cases per year [4]. Furthermore, it should be emphasized that, unlike cervical cancer, the virus affects both sexes equally in the oral cavity and oropharynx [5]. In particular, studies reported an increased incidence rate of HPV-related OPC in the last two decades in Italy [6,7]. Another relevant epidemiological information is the increasing spread of HPV infection in the young population, not only at the genital site but also at the upper aero-digestive tract, due to sexual precocity and promiscuity [3,8]. For this reason, it is important to educate the population, especially the younger generation, about HPV infection and its associated risks, as well as to promote primary and secondary prevention tools, such as vaccination [9] and the PAP test (Papanicolaou test) [10], respectively. Two HPV strains are primarily related to cancer onset: 16 and 18 [11,12]. HPV-16 type was found in about 15% of oral cavity tumors (OSCC) and in about 41% of oropharyngeal tumors (OPSCC); HPV-18 type, however, was identified in about 6% of OSCCs and 0.2% of OPSCCs [13]. Therefore, HPV vaccination is primarily against these two strains. There are different HPV vaccines (two-valent, four-valent, and nine-valent vaccines) and there are different laws regarding it being mandatory or recommended for certain age groups around the world. In particular, the US Food and Drug Administration (FDA) established that it is advisable to vaccinate girls from 12 to 26 years and boys from 13 to 21 years old [14]. Moreover, in 2018, the FDA extended the use of nine-valent vaccines to both men and women aged between 27 and 45 years old too [15,16]. In Italy, to date, this vaccination is recommended and free of charge for young people (males and females) during the 12th year of age, presuming that in most cases sex first takes place at 13 years old.

Therefore, considering the widespread infection and the poor promotion and adherence to vaccination, this study aimed, through a questionnaire, to evaluate the level of knowledge of HPV infection, its implications, and its possible prevention in the Sicilian population.

## 2. Materials and Methods

A prospective observational study was carried out on the Sicilian population through a self-administered questionnaire. The survey lasted from June 2021 to December 2021. The study was approved by Ethics Committee Palermo 1 of University Hospital Paolo Giaccone of Palermo (protocol code 04/2021—28 April 2021). Inclusion criteria were: (1) males and females, (2) 18–80 years old, (3) with consent to participation, and (4) having Italian proficiency. Exclusion criteria were: (1) under 18 years old; (2) not-resident in Sicily; and (3) participants who answered only the first 3 questions. Those who partially answered the questionnaire were also included in the study.

### 2.1. Questionnaire

Our questionnaire consists of 10 multiple-choice questions, written in Italian to avoid the risk of language bias. The questionnaire was designed by the authors based on current literature and similar valid questionnaires [17,18,19,20]. The survey needed only a few minutes to be completed. The survey was self-administered: the questionnaire started only after the participants read the instructions and the purpose of the survey and the implied consent script for participation. In particular, the survey was created on the SurveyMonkey website and disseminated through a link (https://it.surveymonkey.com/r/5W33F5H (accessed on 1 June 2021) shared through the most famous social networks (Facebook, Linkedin, and Instagram) and messaging application (Whatsapp Messenger). The survey was shared among the author’s social media contacts and with the help of Sicilian social media influencers in order to spread the questionnaire to as many and as heterogeneous people as possible. Participation in the survey was voluntary and completely anonymous. To avoid bias derived from multiple answers from the same participant, the survey site prevented the possibility of answering the questionnaire several times from the same device (personal computer, cell phone, or tablet). The order of the questions was always the same and it was not possible to skip some of them: failure to answer a question prevented the continuation of the survey.

The first 3 questions were personal and aimed to categorize the sample on the basis of: #1 gender, #2 age, and #3 own province. Items #4, #5, #6, and #7 investigated the level of knowledge of papillomavirus in terms of risk of developing uterine and oral/oropharyngeal cancer, both in the male and female population. Finally, questions #8, #9, and #10 focused on the papillomavirus vaccine. In particular, item #10 *Are you vaccinated against papillomavirus?* investigated the adherence and/or willingness to receive the vaccination against the Papillomavirus.

### 2.2. Statistical Analysis

The answers were collected on Excel spreadsheets, allowing the analysis of the results presented as numbers and percentages. The collected data were also represented in graphs for immediate and easy access to the results. Moreover, performing binary logistic regression, odd ratios were used to calculate the likelihood of knowledge and awareness about HPV infections and its vaccine in relation to sex (males vs. females). A *p*-value < 0.05 was considered significant.

## 3. Results

According to data collected in 2020 by the National Institute of Statistics (ISTAT), Sicily has 4,833,705 residents. About 60% of the Sicilian population lives in Palermo, Catania, or Messina. Females represent 51.5% of population and the average age is 44.2 years. In regard to educational level, 5.7% have only a secondary education, 31.8% have a high school diploma, and 12.8% have a university degree [21].

A total of 909 people participated in the survey. Based on the exclusion criteria, 65 individuals were excluded. So, a total of 844 individuals were included, and of these, 811 (96.09%) completed the questionnaire. Furthermore, 60.90% of respondents were female and 53.55% were aged between 21 and 30 years. Among female respondents, 66.37% belonged to the 21–30 age group. All the provinces of Sicily were represented in a rather homogeneous way by the sample examined: Agrigento (11.37%), Caltanissetta (10.78%), Catania (10.90%), Enna (9.95%), Messina (9.84%), Palermo (11.85%), Ragusa (10.19%), Syracuse (12.80%), and Trapani (12.32%).

**Figure 1:**
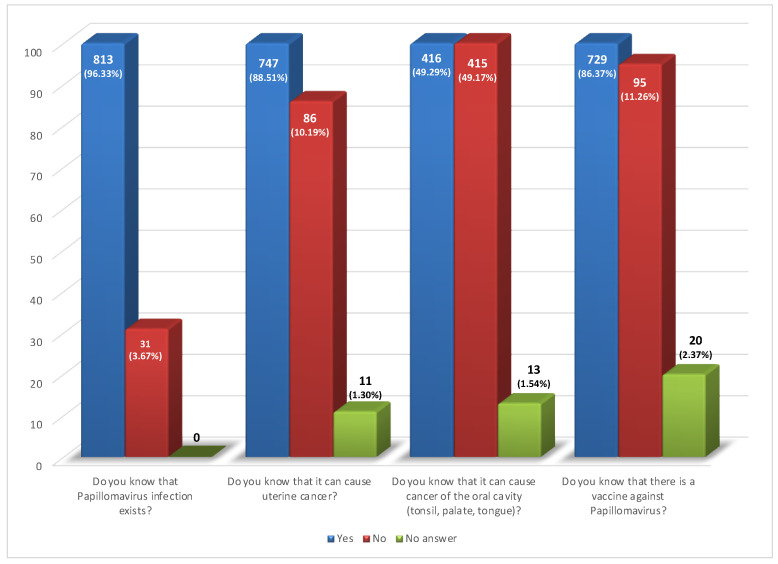
Answers of respondents to the items: #4 Do you know that papillomavirus infection exists?, #5 Do you know that it can cause uterine cancer?, #6 Do you know that it can cause cancer of the oral cavity (tonsil, palate, and tongue)?, #8 Do you know that there is a vaccine against papillomavirus?

The survey found that most of the surveyed population knew about papillomavirus infection (96.33%): in particular, they were female (92.50%), aged between 21 and 30 years (54.61%) (Figure 1), and from the provinces of Syracuse (12.91%) and Trapani (12.67%). Interestingly, for question #6: Do you know that it can cause cancer of the oral cavity (tonsil, palate, tongue)?: 49.29% answered Yes, and 49.17% answered No (Figure 1).

**Figure 2:**
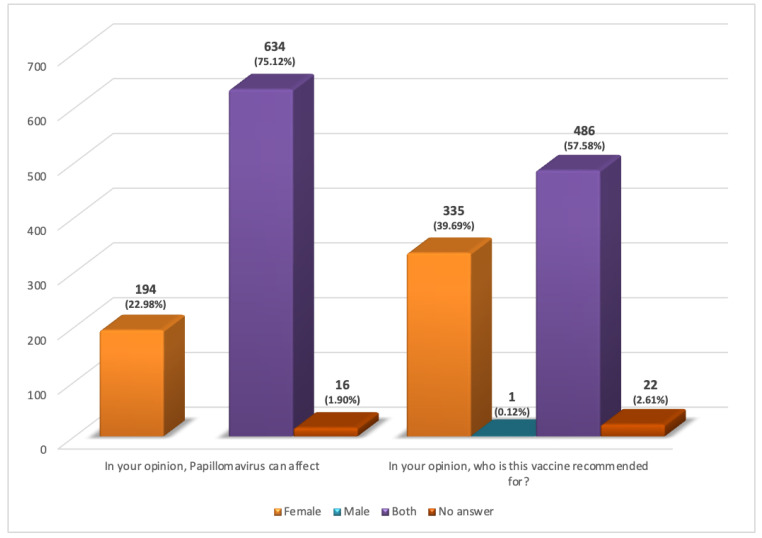
Answers of respondents to the items: #7 In your opinion, papillomavirus can affect? and #9 In your opinion, who is this vaccine recommended for?

194 respondents replied that HPV can only affect females (item #7); 123 were young (between the age of 21 and 40 years old) and 179 were female. However, most of the examined population (634/844) answered this question correctly (Figure 2). Furthermore, 86.37% were aware of the existence of the HPV vaccine but 39.69% believed that this was recommended only to women and 57.58% to both sexes (Figure 1 and Figure 2). The latter group consisted mainly of women (61.73%) and younger people aged between 21 and 30 years (53.29%). In regard to the last question, Are you vaccinated against Papillomavirus?, 34.24% of individuals replied that they were already vaccinated, 42.06% said they wanted to get vaccinated, and 19.79% replied they did not want to get vaccinated (Figure 2). In particular, among the vaccinated, almost all (97.9%) were young (aged between 18 and 40 years) and 69.90% were female. Even the group that wanted to get vaccinated was mainly represented by women (60.28%) and young people aged between 21 and 40 years (70.42%).

**Figure 3:**
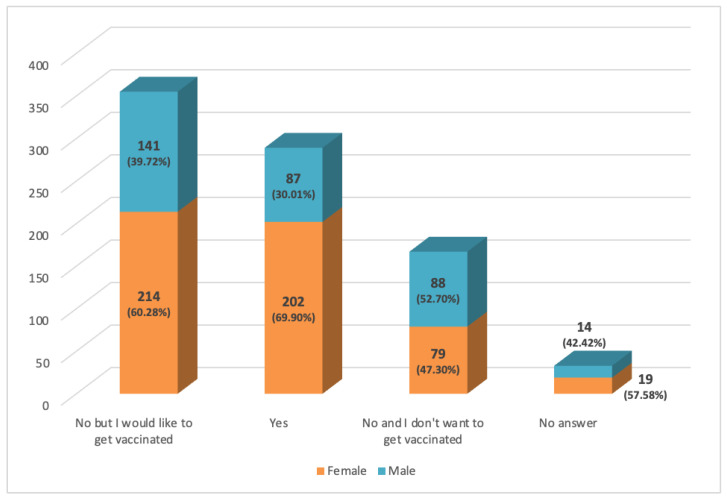
Answers to item #10 Are you vaccinated against papillomavirus?

Most (52.70%) of the respondents who replied that they did not want to vaccinate were male with two age peaks: 21–30 years (26.35%) and 51–60 years (26.35%) (Figure 3). Overall, the most informed province about HPV infection was Syracuse; the province with the highest rate of vaccinated was Trapani (16.81%) (Table 1).

Analyzing the answers in relation to sex of respondents, the logistic regression revealed a not statistically significant value for questions #4 (*p*-value 0.1020) and #7 (*p*-value 0.9185). Results are statistically significant in the case of question #5 (*p*-value 0.001950), #6 (*p*-value 0.04336) and #8 (*p*-value 0.00002497) (Table 2).

## 4. Discussion

Human papillomaviruses can be subdivided into low-risk (LR-HPV) and high-risk (HR-HPV) types. The former is associated with benign lesions, such as papilloma and warts, the latter causes malignant lesions, first of all cervical cancer [22]. Papillomavirus is a DNA virus with oncogenic power due to its expression of two oncoproteins: E6 and E7, which inhibit p53 tumor suppressor proteins and retinoblastoma tumor suppressor proteins, respectively [23,24]. Studies demonstrated that HR-HPV oncoproteins are more efficient than LR-HPV ones in oncogenesis. In particular, in HR-HPV types (e.g., HPV 16 and 18), E6 and E7 oncoproteins can also cause instability of DNA, leading to carcinogenesis [18,22].

To date, HPV is known in the general population for its role in the onset of cervical cancer [1], as also confirmed by our survey, in which 93.33% of respondents knew the virus, especially in the younger population (18–40 years), and 88.51% knew that HPV can cause uterine cancer. We found that only 62.78% of women and 37.22% of men answered correctly, in contrast with results reported by Barnard et al. (93.3% vs. 88.7%) [20] and by Osazuwa-Peters et al. (79.3% vs. 76.1%) [25].

Interesting is the result obtained for question #6 *Do you know that it can cause cancer of the oral cavity?* in which there was a homogeneous distribution of the population between affirmative (49.29%) and negative (49.17%) answers. This result is consistent with the study by Barnard et al. that reported a total of 57.5% correct answers [20]. Additionally, a survey administered in 2014 by the National Cancer Institute (United States) to about 3600 respondents older than 18 years old reported that 60.2% of participants knew that HPV can cause oral cancer [25].

Analyzing the results, females were, overall, more informed about HPV carcinoma both at the uterine (62.78%) and oral (64.42%) sites, in accordance with the literature [26]. In 2019, Kasymova et al. studied the awareness and knowledge of HPV among college students by a self-administered survey. They found that females were more aware than males about HPV infection (97% vs. 88%) and vaccination (95% vs. 76%) [19]. Similar results were achieved in another study, with the prevalence of high knowledge about HPV and its vaccination among females more than males, respectively, 92.4% vs. 82.9% and 75.8% vs. 56.2% [20].

Today, the detection of this virus in the oral cavity and oropharynx is very frequent, with a prevalence ranging between 40 and 90% according to studies [27,28]. In particular, the main factors related to high incidence of HPV infection in these sites are: aged between 40 and 60 years, male, white race, numerous sexual partners, and oral sex [29]. This infection can cause benign (papilloma) and malignant (carcinoma) lesions. The young adult population is most often affected by HPV-related OPCs and, frequently, the involvement of the latero-cervical lymph nodes is rather early [30,31]. Therefore, it is essential to spread the knowledge of HPV infection and its consequences among both adults and adolescents. Indeed, prevention is the best weapon to fight HPV infection and HPV-related OPC. A study carried out in the United States (US) in 2014 assessed the level of knowledge of HPV, HPV-related tumors, and HPV vaccines in the uninsured adult population and observed a lack of knowledge of the oncogenic properties of the virus and the existence of a vaccine to prevent it. In addition, it identified the main causes of low vaccination coverage: lack of knowledge of the vaccine or the risks related to certain sexual behavior (number of sexual partners and oro-genital sexual practices), failure of general practitioner to recommend vaccination, and high cost of the vaccine [26]. In our sample, 86.37% of respondents were aware of the existence of the HPV vaccine, mostly females; conversely, among those who ignored the vaccine, the majority were males (58.95%).

To date, there are several types of vaccines: (1) two-valent vaccine, targeting HPV types 16 and 18; (2) four-valent vaccine, targeting HPV types 6, 11, 16, and 18; and (3) nine-valent vaccine, targeting also HPV types 31, 33, 45, 52, and 58. If administered prior to HPV exposure, the two-valent vaccine reduces onset of cervical precancerous lesions and oral infection by HPV types 16 and 18 in about 90% of cases. In the same way, quadrivalent vaccine prevents HPV infections and cervical and oral precancerous lesions caused by HPV 6, 11, 16, or 18 in around 90% of cases. Nine-valent vaccine is more efficient and is shown to significantly reduce the risk of developing cervical cancer (risk reduced to about 5% if administered before HPV exposure) and other HPV-related premalignant and malignant lesions, such as penile, anal, vulvar, vaginal, and oropharyngeal ones (risk reduced by about 80%) [32,33,34]. In our survey, only 289 respondents (34.24%) were vaccinated, and the majority of these were female. These data are consistent with a study by Fishman et al. [18], who demonstrated that high knowledge about HPV infection and risks was not related to level of vaccination. Moreover, in regard to vaccination rate based on sex, a study by Barnard et al. reported that there are more vaccinated females than vaccinated males (47.3% vs. 15.8%) [20].

The most alarming data, however, is that 19.79% of respondents said they did not want to get vaccinated and 52.70% were male, confirming the common opinion that HPV is responsible only for cervical cancer which, therefore, does not affect men. In fact, 22.98% of respondents replied that HPV affects females at item #7. Data are consistent with current literature [20].

These answers, therefore, highlight the lack of knowledge of a growing widespread and dangerous infection among young people and, above all, do not consider the epidemiological data now established: HPV-related OPC affects men more often [35,36]. Another important finding that emerged from our study is that 39.69% of respondents, of whom 60% were women, claimed that the vaccine was recommended only for women. Actually, as previously written, to date in Italy, this vaccination is recommended and free of charge for younger people (males and females) during the 12th year of age, whereas in most cases the first instance of sexual intercourse occurs at the age of 13.

Therefore, from the analysis of the results obtained, this study shows incomplete knowledge of HPV: on the one hand, in fact, most of the assessed population proved to know the virus, the HPV-related cervical cancer, and the existence of a vaccine; on the other hand, however, a significant portion of the respondents ignored the effects of the virus at the oral and oropharyngeal sites and the indication for vaccination also for males.

However, the study has some limitations: (1) although the internet and social networks are now widely used, there is a poorer and illiterate part of the population that the study could not include and assess; (2) all provinces of Sicily were sufficiently represented in the sample examined, even though the percentage of responses was not correlated with the actual population of each province; (3) in item #6, the expression “oral cavity” also included oropharyngeal structures to facilitate the understanding of the question to the general population; and (4) results are based on self-reported data because we cannot prove in any way the truthfulness of the answers. However, to reduce or avoid the risk of social desirability bias in answering, the survey was anonymous.

## 5. Conclusions

Oral and oropharyngeal HPV-related cancers are a growing frequent finding, especially in young adults. A prevention campaign, both primary and secondary, is therefore essential and necessary, addressed not only to women in certain age groups, but also to the entire young-adult population. Our study revealed an incomplete and “misleading” knowledge about papillomavirus, HPV infection, and HPV vaccination, which implies the need to educate the population about the risks that certain voluptuous habits can provoke, with the help of general practitioners, schools, social media, and social networks. In conclusion, the main message to take home is: better safe than sorry!

## Figures and Tables

**Table 1:** HPV survey on Sicilian population.

Items	Multiple Answers	N° (%)	
1 **Sex**	Female	514 (60.90)	
	Male	330 (30.10)	
2 **Age**	18–20 yo	39 (4.62)	
	21–30 yo	452 (53.55)	Female: 300 (66.37 Male: 152 (33.63)
	31–40 yo	144 (17.06)	
	41–50 yo	92 (10.90)	
	51–60 yo	88 (10.43)	
	>61 yo	29 (3.44)	
3 **Where do you live?**	Province of Agrigento	96 (11.37)	
	Province of Caltanissetta	91 (10.90)	
	Province of Catania	92 (10.90)	
	Province of Enna	84 (9.95)	
	Province of Messina	83 (.84)	
	Province of Palermo	100 (11.85)	
	Province of Ragusa	86 (10.19)	
	Province of Siracusa	108 (12.80)	
	Province of Trapani	104 (12.32)	
4 **Do you know that papillomavirus infection exists?**	Yes	813 (96.33)	Female: 752 (92.50)
			Male: 61 (7.50) 18–20 yo: 36 (4.43) 21–30 yo: 444 (54.61) 31–40 yo: 140 (17.22) 41–50 yo: 89 (10.95) 51–60 yo: 81 (9.96) >61 yo: 23 (2.83) Siracusa *: 105 (12.91) Trapani *: 103 (12.67)
	No	31 (3.67)	Female: 22 (70.97) Male: 9 (29.03) 18–20 yo: 3 (9.68) 21–30 yo: 8 (25.81) 31–40 yo: 4(12.90) 41–50 yo: 3 (9.68) 51–60 yo: 7 (22.58) >61 yo: 6 (19.35) Palermo *: 10 (32.26)
5 **Do you know that papillomavirus can cause uterine cancer?**	Yes	787 (88.51)	Female: 469 (62.78) Male: 278 (37.22)
	No	86 (10.19)	Female: 39 (45.35) Male: 47 (54.65)
	No answer	11 (1.30)	
6 **Do you know that papillomavirus can cause cancer of the oral cavity (tonsil, palate, and tongue)?**	Yes	416 (49.29)	Female: 268 (64.42) Male: 148 (35.58)
	No	415 (49.17)	Female: 239 (57.59) Male: 176 (42.41)
	No answer	13 (1.54)	
7 **In your opinion, papillomavirus can affect:**	Female	194 (22.98)	Female: 179 (92.27) Male: 15 (7.73) 18–20 yo: 10 (5.15) 21–30 yo: 91 (46.91) 31–40 yo: 32 (16.50) 41–50 yo: 27 (13.92) 51–60 yo: 26 (13.40) >61 yo: 8 (4.12) Agrigento *: 28 (14.43)
	Both	634 (75.12)	Female: 582 (91.80) Male: 52 (8.20) 18–20 yo: 26 (4.10) 21–30 yo: 355 (55.99) 31–40 yo: 111 (17.51) 41–50 yo: 63 (9.94) 51–60 yo: 59 (9.31) >61 yo: 20 (3.15) Siracusa *: 83 (13.09)
	No answer	16 (1.90)	
8 **Do you know that there is a vaccine against papillomavirus?**	Yes	729 (86.37)	Female: 465 (63.79) Male: 264 (36.21)
	No	95 (11.26)	Female: 39 (41.05) Male: 56 (58.95)
	No answer	20 (2.37)	
9 **In your opinion, who is this vaccine recommended for?**	Female	335 (39.69)	Female: 201 (60.00) Male: 134 (40.00) 18–20 yo: 13 (3.88) 21–30 yo: 182 (54.33) 31–40 yo: 64 (19.11) 41–50 yo: 33 (9.85) 51–60 yo: 32 (9.55) >61 yo: 11 (3.28) Trapani *: 44 (13.13)
	Male	1 (0.12)	Female: 1 (100) 31–40 yo: 1 (100)
	Both	486 (57.58)	Female: 300 (61.73) Male: 186 (38.27) 18–20 yo: 23 (4.73) 21–30 yo: 259 (53.29) 31–40 yo: 77 (15.84) 41–50 yo: 57 (11.73) 51–60 yo: 53 (10.91) >61 yo: 17 (3.50) Siracusa *: 68 (13.99) Palermo *: 61 (12.55)
10 **Are you vaccinated against papillomavirus?**	Yes	289 (34.24)	Female: 202 (69.90) Male: 87 (30.01) 18–20 yo: 26 (9.00) 21–30 yo: 234 (80.97) 31–40 yo: 23 (7.96) 41–50 yo: 2 (0.69) 51–60 yo: 3 (1.04) >61 yo: 1 (0.34) Trapani *: 48 (16.81)
	No and I do not want to get vaccinated	167 (19.79)	Female: 79 (47.30) Male: 88 (52.70) 18–20 yo: 3 (1.79) 21–30 yo: 44 (26.35) 31–40 yo: 25 (14.97) 41–50 yo: 32 (19.16) 51–60 yo: 44 (26.35) >61 yo: 19 (11.38) Palermo *: 38 (22.75)
	No but I would like to get vaccinated	355 (42.06)	Female: 214 (60.28) Male: 141 (39.72) 18–20 yo: 6 (1.69) 21–30 yo: 158 (44.51) 31–40 yo: 92 (25.92) 41–50 yo: 54 (15.21) 51–60 yo: 37 (10.42) >61 yo: 8 (2.25) Palermo *: 49 (13.80)
	No answer	33 (3.91)	
	**TOTAL**	844 (100)	

yo: years old; * provinces with the highest number of answers.

**Table 2:** Logistic regression results.

Question	Odd	Chi-Squared (X^2^)	*p*-Value
#4 Do you know that papillomavirus infection exists?	1.1333	2.6739	0.1020
#5 Do you know that papillomavirus can cause uterine cancer?	1.2051	9.5958	0.001950
#6 Do you know that papillomavirus can cause cancer of the oral cavity (tonsil, palate, and tongue)?	0.7364	4.0813	0.04336
#7 In your opinion, papillomavirus can affect:	0.6441	0.01047	0.9185
#8 Do you know that there is a vaccine against papillomavirus?	1.4359	17.7669	0.00002497

## Data Availability

Not applicable.
